# Prevalence of Hypertension and Its Determinants Among Adult People Living in Riverine Island, Sirajganj, Bangladesh: A Cross‐Sectional Study

**DOI:** 10.1002/hsr2.70527

**Published:** 2025-02-24

**Authors:** Safayet Jamil, Mohammad Shahangir Biswas, Habib Mohammad Ali, Neeru Chaudhary, Chandrima Chatterjee, Md. Emdadul Hasan Mukul, Victor Abiola Adepoju, Alauddin Chowdhury Abm

**Affiliations:** ^1^ Faculty of Health, Education and Life Science Birmingham City University Birmingham UK; ^2^ Department of Public Health Daffodil International University Dhaka Bangladesh; ^3^ Department of Public and Community Health, Faculty of Medicine and Health Sciences Frontier University Garowe Puntland Somalia; ^4^ Department of Biochemistry and Biotechnology, School of Biomedical Science Khwaja Yunus Ali University Sirajganj Bangladesh; ^5^ Department of Biochemistry and Biotechnology University of Science and Technology Chittagong Chattogram Bangladesh; ^6^ Department of Media Studies and Journalism University of Liberal Arts Bangladesh (ULAB) Dhaka Mohammadpur Bangladesh; ^7^ Delhi Pharmaceutical Sciences and Research University New Delhi India; ^8^ Department of Health Management and Systems Sciences University of Louisville Louisville Kentucky USA; ^9^ Department of Pharmacy University of Rajshahi Rajshahi Bangladesh; ^10^ Department of HIV and Infectious Diseases Jhpiego Nigeria, An Affiliate of John Hopkins University Abuja Nigeria

**Keywords:** Bangladesh, cardiovascular disease, high blood pressure, hyperpiesis, hypertension

## Abstract

**Background and Aims:**

Assessing the prevalence of hypertension and associated determinants in riverine island populations is crucial due to their unique socio‐environmental characteristics and restricted healthcare access, which increase sensitivity to hypertension‐related problems and should lead targeted interventions. So, this study investigates the concerning issue of hypertension, focusing on its prevalence and influential factors among adults in Sirajganj's Riverine Island.

**Methods:**

By employing a cross‐sectional approach, the research utilized a structured questionnaire. A total of 309 people took part in this study via face‐to‐face interviews by following convenience sampling technique. The investigators performed frequency analysis for getting prevalence of HTN and other variable's percentage and binary logistic regression analysis for getting associated determinants. Analysis was conducted by using a statistical package for social sciences (SPSS) version 25.

**Results:**

The prevalence of hypertension was 41% among all individuals. People who were illiterate were 1.23 times more hypertensive. Some key risk factors were discovered, including smoking (*p* = 0.04), adding salt to meals (*p* < 0.001), eating fatty or fat‐type foods (*p* < 0.001), sleeping late (*p* < 0.001), worrying about their lifestyle and future (*p* < 0.001), and not exercising (*p* = 0.03). Males were 1.43 times more hypertensive (AOR = 1.43, 95% CI: 1.17−2.0, *p* = 0.002) than females. Smokers were 4.21 times more hypertensive (AOR = 4.21, 95% CI: 1.12−17.13, *p* = 0.04) than nonsmokers. Consumers oil/fat with food were 2.18 times more hypertensive (AOR = 2.18, 95% CI: 1.78−2.54, *p* < 0.001) than others. Respondents who were worried about their lifestyle and future were 3.38 times more hypertensive (AOR = 3.38, 95% CI: 1.27−6.01, *p* = 0.001) than others.

**Conclusion:**

People should consume a healthy diet and refrain from smoking. Regular physical activity and going to bed early are recommended. Medical camps and workshops should be held on riverine islands to promote a healthy lifestyle and prevent hypertension.

## 1 Introduction

Hypertension (HTN) is a serious medical and public health issue. It remains a leading global health crisis, with recent estimates suggesting an alarming rise in associated mortalities and morbidities. Projections indicate a steep incline in adult HTN prevalence, particularly in South Asian regions, necessitating urgent public health attention [1]. In Bangladesh, the impact of HTN is profound, as this affects up to 65% of the elderly, with varying degrees of prevalence across different regions [2].

In the not‐too‐distant future, HTN and other associated illnesses (diabetes, heart disease, and stroke) will be the main causes of death, disability, and medical costs. Screening and treatment of high blood pressure are the key preventative measures for reducing death and disability from cardiovascular diseases. Metabolic syndromes, compounded by lifestyle factors like diet and physical inactivity, are significantly influencing the pathogenesis of HTN in the local context of Bangladesh [1]. This epidemiological shift toward noncommunicable diseases in Bangladesh, especially HTN, underscores the critical need for focused studies like this [3]. Many countries around the world are dealing with the double burden of disease, and the South Asian region is no exception. The total mortality rate has decreased significantly during the last two decades [3]. However, the incidence of deaths from chronic illnesses, particularly the “fatal four” of diabetes, chronic lung disease, cancer, and cardiovascular disease, is on the rise. HTN is a key cause of one of them, namely cardiovascular disease [2].

Several investigations in South Asia have been undertaken to determine the prevalence of HTN [3, 4, 5]. Researchers have also looked at the various regions of Bangladesh to better understand the epidemiology of HTN in this country. The Bangladesh Demographic and Health Survey (BDHS) data sets was examined, which comprised persons aged 35 and older who were surveyed between July and December 2011 [5]. The BDHS used a multistage stratified cluster sampling design to choose 8835 household members, with an overall response rate of 89.2% [6]. The sample was reduced to 7876 participants after nonrespondents and missing data were eliminated. When age was taken into account, the prevalence of prehypertension and HTN was 27.1% and 24.4%, respectively. Only 31.4% of people with HTN had their blood pressure under control, despite the fact that 50.1% were aware of their condition and 41.2% were receiving treatment. The poor and wealthy households dealt with HTN in quite diverse ways [7, 8].

In Bangladesh, one in every four people has HTN, and nothing is known about it or done to manage it. To lessen the rising burden of HTN, diagnostic and treatment approaches must be improved [1]. According to Khanam et al., in a study of elderly people in the Matlab Health and Demographic Surveillance Area, 50% of elderly adults in rural Bangladesh had HTN, but only 26% kept their blood pressure under control [6]. Another study was carried out in rural Bangladesh. The Matlab, Abhoynagar, and Mirsarai rural demographic surveillance sites provided the study population. The lowlands and riverine regions make up the majority of rural Bangladesh. The delivery of public healthcare is fairly consistent across the country. Participants in the study had to be at least 25 years old. In 2009, each site conducted a 2‐month cross‐sectional survey. HTN was found in 13.67% of the population. Despite the fact that village doctors diagnose 40% of HTN patients, their treatments are associated with higher rates of drug nonadherence [5, 7]. More research should be conducted to investigate how village doctors treat HTN. Men, young individuals, and people with low levels of education should be prioritized. As a result, it is critical for health programs to educate individuals about the importance of taking antihypertensive medicine on a continuous basis [9].

The epidemiology of HTN in Bangladesh is governed by several factors, including awareness, health literacy, correct and prompt diagnosis, proper prescription of drugs, and adherence to therapy. This study investigated the frequency of HTN among adult people living on Bangladesh's riverine islands. It also investigated the related risk factors for the progression of HTN in the defined group.

## 2 Materials and Methods

### 2.1 Study Design and Settings

Applying the quantitative research approach, this study was a survey with a cross‐sectional design. This research was carried out during the dates of February 22 and March 31, 2023. The purpose of this investigation was explicitly set out in detail on the very first page of the questionnaire. This manuscript was prepared following the guidelines of the STROBE checklist [10]. People who lived on Riverine Island were 19–50 years old and were willing to engage in the study were required to meet all three inclusion criteria in order to be considered for the study. A person was ineligible for participation in the survey if they met all three of the following criteria: (a) they did not live in the riverine island area as a permanent residence; (b) they provided an incomplete answer; and (c) they were older than 50.

### 2.2 Study Population, Sampling Technique, and Sample Size

The study was conducted in the riverine landscape of Chauhali Upazila, a locality within Sirajganj, Bangladesh, known for its unique health challenges. Chauhali Upazila has a population of approximately 134,026 [11]. The following formula was used to calculate the sample size [12] –$n=\frac{z^{2}\mathrm{pq}}{d^{2}}\;*\;\text{design effect}$; Where, *n* = desired sample size; *z* = 1.96 (95% confidence interval), p = population proportion (considering 50%), *q* = 1–*p*, *d* = precision level (5%), effect of design = 2.0, and adjusting 10.0% of nonresponse. Upon calculation, we determined an initial sample size of 275, which was eventually expanded to 331 to improve the robustness of the study. Utilizing a convenience sampling strategy, a total of 331 responses were received and included in the analysis. For some incomplete responses, finally, we used 309 data, excluding 22 responses.

### 2.3 Questionnaire Development, Variables, and Data Collection

The questionnaire featured 17 closed‐ended questions. It was divided into three sections: questions pertaining to demographic factors, questions pertaining to lifestyle patterns, and questions pertaining to HTN. The demographic variables section included age, sex, marital status, educational status, occupation, and socioeconomic status. The lifestyle patterns section included exercise records, smoking behavior, adding salt to their food, consuming oil/fat type foods, sleeping late at night, and worried about their lifestyle and their future. All variables of section two were measured by two categories including “yes” and “no.” “Adding salt to your food” means participants may add dietary salt in their daily meal which can cause high blood pressure and cardiovascular disease [13]. “Do you take food with oil/fat type” means participants may take oily food or fried/fast food which may cause cardiovascular disease or HBP [11]. “Going to bed late at night” means participants may go to bed at late night which may cause may health complications [14]. And “Worried about your lifestyle and your future” means participants may have fear and dissatisfaction about their life which can effect on their mind and their heart [7]. Section 3 was contained some HTN‐related questions including having HTN, knowledge about HTN, causes of HTN, and timeline of suffering from HTN. The prevalence of HTN was expressed by “Have you been diagnosed with hypertension?.” Through a literature review, all of the questions were derived from previously published research studies [3, 4, 5]. Two professionals evaluated the questionnaire to ensure that it was accurate in terms of its content. The questions were moderated after they had been checked. After an initial round of testing with 25 participants, the responses from those participants were subsequently excluded from the primary analysis. The questionnaire was created to be filled out in English. Following a brief period of moderation, the face‐to‐face method of final data gathering was initiated [11]. There was a total of four research assistants assigned to the data collection process. After that, they received 2 days' worth of training on the process of data gathering. They began by providing the respondents with an explanation of the purpose of this research and collecting responses from those who were willing to participate. This ensured that all of the regulations and procedures were carried out correctly. In the end, data were gathered from the participants by conducting interviews with them face‐to‐face.

### 2.4 Bias

As a self‐ministered questionnaire was used in this study, the response bias may be happened.

### 2.5 Data Analysis and Software

The defined guidelines provided by Assel et al. were followed in the data processing and interpretation of the findings of the study [15]. SPSS 25.0 version was used during data analysis. Frequency analysis was done for getting the percentage of all demographic variables, lifestyle patterns variables, and having HTN and knowledge of HTN. Binary logistic regression analysis was performed for identifying the associated risk factors of HTN. A *p*‐value less than 0.05 was considered to be statistically significant.

### 2.6 Ethical Consideration

This study was approved by the Khwaja Yunus Ali University Ethical Grant Committee by letter No. KYAU/DEAN/EGC/2024/010. Also, everything was done in accordance with the World Medical Declaration of Helsinki [16].

## 3 Results

### 3.1 Demographic Status of the Participants

Table 1 shows the demographic makeup of the study participants. Notably, 39.16% of the study participants were 31−40 age year old. 35.28% of the participants were 41−50 years old and 25.56% participants were 19−30 years old. Most of the participants were male (64.08%). Maximum participants (87.32%) were married in their marital status.

**Table 1:** Demographic variables (*N* = 309).

Variables	*N* (%)
Age	
19−30	79 (25.56%)
31−40	121 (39.16%)
41−50	109 (35.28%)
Sex	
Male	198 (64.08%)
Female	111 (35.92%)
Marital status	
Married	242 (78.32%)
Single	67 (21.68%)
Educational status	
Illiterate	138 (44.66%)
Primary and secondary	122 (39.48%)
Higher secondary and above	49 (15.86%)
Occupation	
Van driver	29 (9.39%)
Fisherman	116 (37.54%)
Boatman	77 (24.92%)
Farmer	51 (16.50%)
Businessman	16 (5.18%)
Job holder	20 (6.47%)
Socioeconomic status	
< 10,000 BDT (Bangladeshi taka)	196 (64.43%)
> 10,000 BDT	113 (36.57%)

Abbreviation: BDT = Bangladesh Taka.

A total of 44.66% of the participants were illiterate. 39.48% of participants completed their primary and secondary education, and 15.86% of participants completed their higher secondary and above. Occupation of the participants of this study were Van drivers (9.39%), Fishermen (37.54%), Boatmen (24.92%), Farmers (16.50%), Businessmen (5.18%), and Job holders (6.47%). Most of the participant's (64.43%) income status was below 10,000 BDT, and 36.57% of participant's income status was above 10,000 BDT.

### 3.2 Lifestyle Pattern of Participants

Table 2 demonstrates the lifestyle pattern of participants. Only 17.15% participants took exercise regularly. According to this study, 64.4% participants were smoker. Total 90.94% of the participants added salt to their food in every meal. 85.76% of the participants took food with oil/fat type food in every meal. 31.72% participants went to bed at late night. Most of the participants (78.96%) worried about their lifestyle and their future.

**Table 2:** Lifestyle of participants.

Variables	*N* (%)
Taking exercise (everyday)	
Yes	53 (17.15%)
No	256 (82.85%)
Having smoking behavior	
Yes	199 (64.40%)
No	110 (35.60%)
Adding salt to your food (every meal)	
Yes	281 (90.94%)
No	28 (9.06%)
Do you take food with oil/fat type? (every meal)	
Yes	265 (85.76%)
No	44 (14.24%)
Going to bed at late night (every night)	
Yes	98 (31.72%)
No	211 (68.28%)
Worried about your lifestyle and your future	
Yes	244 (78.96%)
No	65 (21.04%)

**Figure 1:**
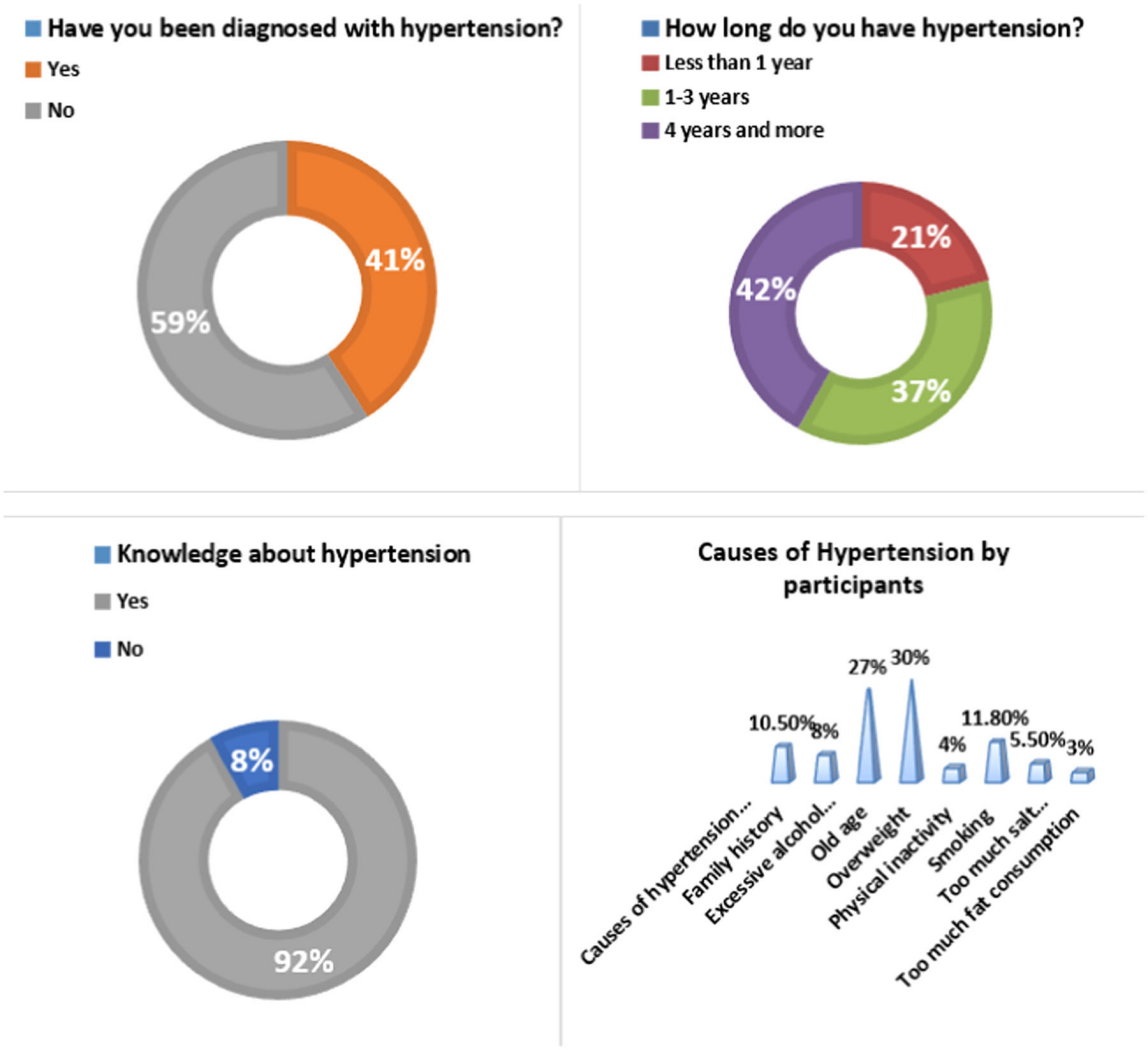
Variables related to hypertension and knowledge.

Figure 1 shows factors influencing HTN. In this study, a total of 41% of the participants had HTN. Most of the participants were hypertensive for 4 years or more. Twenty‐one percent of participants had HTN for less than 1 year. Ninety‐two percent of participants had knowledge about HTN. Maximum 30% participants knew that overweight is the main cause of HTN. Twenty‐seven percent of participants knew that old age was the cause of HTN. 11.80% participants knew that smoking is the cause of HTN. 10.5%, 8%, 5.5%, 4%, and 3% of participants knew that HTN is caused by family history, excessive alcohol consuming, too much salt consuming, physical inactivity, and too much fat consumption, respectively.

### 3.3 Association Between HTN and Demographic Characteristics

Table 3 represents the association of demographic status with HTN. Participants who were 31−45 years old had 72% lower chances with HTN (OR: 0.32; CI: 0.20−0.49; *p* < 0.001) than 45 years old and above. Males had 1.43 times more HTN (OR: 1.43; CI: 1.17−2.0; *p* = 0.002) than females. Illiterate participants were 1.23 times more hypertensive (OR: 1.23; CI: 1.18−1.60; *p* < 0.001). Businessmen were 1.15 times more hypertensive (OR: 1.15; CI: 0.99−1.52; *p* = 0.001) than job holders.

**Table 3:** Finding potential factors between demographic variables and hypertension by binary logistic regression analysis.

Variables	OR (95% CI)	*p* value
Age		
19−30	0.93 (0.89–1.02)	0.654
31−45	0.32 (0.20‐0.49)	< 0.001
45 to above	1	1
Sex		
Male	1.43 (1.17−2.0)	0.002
Female	1	1
Marital status		
Married	1.09 (0.87−1.20)	0.851
Single	1	1
Educational status		
Illiterate	1.23 (1.18–1.60)	< 0.001
Primary and secondary	1.88 (0.80–4.23)	0.091
Higher secondary and above	1	1
Occupation		
Van driver	4.55 (0.49–36.45)	0.134
Fisherman	1.01 (0.95–2.77)	0.424
Boatman	0.90 (0.99–2.30)	0.331
Farmer	3.95 (0.42–29.76)	0.112
Businessman	1.15 (0.99–1.52)	0.001
Job holder	1	1
Socioeconomic status		
< 10,000 BDT	1.03 (0.90–1.20)	0.771
> 10,000 BDT	1	1

### 3.4 Association Between HTN and Lifestyle Pattern

Table 4 demonstrates the association of lifestyle patterns with HTN. The study found that participants who smoked were 4.21 times more likely to have HTN, underscoring the impact of lifestyle choices (OR: 4.21; CI: 1.10−17.13; *p* = 0.04) than nonsmoker. Participants who consumed salt in their food (every meal) had 3.30 times more HTN (OR: 3.30; CI: 2.51−5.89; *p* < 0.001). Participants who took food with oil/fat type in every meal had 2.18 times more HTN (OR: 2.18; CI: 1.78−2.54; *p* < 0.001). Participants who did late night were 1.11 times more hypertensive (OR: 1.11; CI: 1.09−1.27; *p* < 0.001). Participants who worried about their lifestyle and their future had 3.38 times more HTN (OR: 3.38; CI: 1.27−6.01; *p* = 0.001).

**Table 4:** Finding potential factors between lifestyle of participants and hypertension by binary logistic regression analysis.

Variables	OR (95% CI)	*p* value
Taking exercise		
Yes	0.96 (0.93−0.99)	0.03
No	1	1
Having smoking behavior		
Yes	4.21 (1.10−17.13)	0.04
No	1	1
Adding salt to your food		
Yes	3.30 (2.51−5.89)	< 0.001
No	1	1
Do you take food with oil/fat type?		
Yes	2.18 (1.78−2.54)	< 0.001
No	1	1
Going to bed at late night		
Yes	1.11 (1.09−1.27)	< 0.001
No	1	1
Worried about your lifestyle and your future		
Yes	3.38 (1.27−6.01)	0.001
No	1	1

## 4 Discussion

The discussion contextualizes HTN as not just a global health crisis but as a localized public health emergency, particularly in the riverine island of Sirajganj. It is important to investigate the prevalence of and its risk factors among this specific demographic to tailor effective health strategies. In this study, we investigated the relationship between HTN and demographic characteristics and lifestyle habits among adult residents of Bangladesh's Riverine Islands.

Demographic factors are important in determining the prevalence of HTN. The age distribution of study participants, primarily between 31 and 50, sheds light on the age group most at risk of HTN, and this not only echoes global patterns but also reveals local nuances. This age distribution implies that the risk of HTN increases with age, which is consistent with prior research [8, 17]. Furthermore, being male was found to be a substantial risk factor for HTN, with males having 1.43 times the odds of HTN as females. This gender disparity in HTN prevalence has been documented in a variety of populations [18, 19]. Another key demographic characteristic associated with HTN is education level. Participants who were illiterate were shown to have a higher risk of HTN than those who had completed elementary and secondary school. Other research has found that low educational attainment is related to poor health literacy and harmful behaviors, increasing the risk of HTN [20, 21, 22, 23]. In our investigation, occupation and socioeconomic position were not found to be substantially linked with HTN. The study highlights the need for more targeted research into occupational health risks, particularly in underrepresented groups like van drivers and fishermen. To investigate the association between occupation, socioeconomic status, and HTN more thoroughly, higher sample sizes are required [24, 25, 26, 27, 28, 29]. The result highlights how lifestyle choices, particularly in this riverine context, play a pivotal role in both the onset and progression of HTN. This finding further supports the need for community‐based fitness programs and health campaigns to promote regular exercise. Regular physical activity has been proven in numerous studies to lower the risk of HTN [26, 30, 31]. As a result, encouraging and supporting physical activity should be a top priority in HTN prevention efforts on the riverine islands. In our study, smoking, adding salt to food, eating oily or fat‐type foods, sleeping late, and worrying about lifestyle and the future were all found to be major risk factors for HTN. Previous research has shown that smoking, excessive salt intake, unhealthy dietary habits, poor sleep quality, and psychological stress all have a negative impact on blood pressure regulation and the development of HTN [32, 33, 34]. According to research in Bangladesh, 18.8% of people had cardiovascular disease for taking oily and fast food where oily food consumers were 2.18 times more hypertensive in our study [11]. In 2023, Odame et al. reported that people who were depressed and dissatisfied with their lifestyle were more hypertensive, which is similar to this study [35]. Additionally, our study reported that late‐night sleeping is significantly associated with HTN. Going to bed late can lead to HTN by disrupting circadian rhythms, reducing sleep duration, and altering stress responses [36]. The investigators propose the implementation of culturally sensitive and accessible lifestyle intervention programs, including localized smoking cessation initiatives and dietary workshops tailored to the community's needs.

There are certain limitations in our study, including the sampling method and the cross‐sectional design, which may influence the breadth and depth of our conclusions. The cross‐sectional nature indicates that the study could not establish causal relationships, urging caution in interpretation. Longitudinal studies could yield deeper insights, especially in establishing temporal relationships between risk factors and HTN onset, and should be prioritized in future research. Conducting this study within a specific geographic locale, while beneficial for focused insights, potentially limit the unique demographic characteristics of our study population and might not mirror those of other regions, underscoring the need for localized approaches in HTN research. The investigators advocate for future research to embrace a more diverse demographic scope, which could provide a more holistic understanding of the impact of HTN across varied populations. Given the study's limited time frame, extending the duration in future research could provide much more detailed insights into HTN trends over time.

## 5 Conclusion

In conclusion, the study provides valuable insight on the prevalence and multifaceted risk factors of HTN, paving the way for informed public health strategies and interventions. The findings emphasize the necessity of taking demographic factors and lifestyle behaviors into account when assessing the burden of HTN and developing effective preventative methods. We advocated for a holistic approach to health, emphasizing dietary modifications and smoking cessation as key preventive measures against HTN. We propose that future studies should focus on genetic, environmental, and socioeconomic factors that underlie HTN in riverine populations.

## Author Contributions

**Safayet Jamil:** conceptualization, methodology, software, data curation, investigation, formal analysis, writing – original draft, writing – review and editing, validation. **Mohammad Shahangir Biswas:** conceptualization, methodology, supervision, formal analysis, investigation, writing – original draft, writing – review and editing, project administration, data curation, software, visualization. **Habib Mohammad Ali:** writing – review and editing, conceptualization. **Neeru Chaudhary:** writing – review and editing, conceptualization. **Chandrima Chatterjee:** conceptualization, writing – review and editing. **Md Emdadul Hasan Mukul:** conceptualization, writing – review and editing. **Victor Abiola Adepoju:** conceptualization, writing – review and editing. **Alauddin Chowdhury ABM:** conceptualization, writing – review and editing.

## Ethics Statement

This study was approved by the Khwaja Yunus Ali Ethical Grant Committee by letter No. KYAU/DEAN/EGC/2024/010. Also, everything was done in accordance with the World Medical Declaration of Helsinki (Kabir et al. 2022).

## Consent

Informed consent was taken from all the respondents.

## Conflicts of Interest

The authors declare no conflicts of interest.

## Transparency Statement

The lead author, Mohammad Shahangir Biswas, affirms that this manuscript is an honest, accurate, and transparent account of the study being reported, that no important aspects of the study have been omitted, and that any discrepancies from the study as planned (and, if relevant, registered) have been explained.

## Data Availability

Since no new data were created or analyzed in this study, data sharing is not applicable to this article; all of the data underlying the results presented are available in the article.
